# The Economic Burden of Cancers on Indian Households

**DOI:** 10.1371/journal.pone.0071853

**Published:** 2013-08-12

**Authors:** Ajay Mahal, Anup Karan, Victoria Y. Fan, Michael Engelgau

**Affiliations:** 1 School of Public Health and Preventive Medicine, Monash University, Melbourne, Australia; 2 Public Health Foundation of India, New Delhi, India, and University of Oxford, Oxford, United Kingdom; 3 Center for Global Development, Washington, District of Columbia, United States of America; 4 South Asia Human Development Unit, The World Bank, Washington, District of Columbia, United States of America; Davidoff Center, Israel

## Abstract

We assessed the burden of cancer on households’ out-of-pocket health spending, non-medical consumption, workforce participation, and debt and asset sales using data from a nationally representative health and morbidity survey in India for 2004 of nearly 74 thousand households. Propensity scores were used to match households containing a member diagnosed with cancer (i.e. cancer-affected households) to households with similar socioeconomic and demographic characteristics (controls). Our estimates are based on data from 1,645 households chosen through matching. Cancer-affected households experienced higher levels of outpatient visits and hospital admissions and increased out-of-pocket health expenditures per member, relative to controls. Cancer-affected households spent between Indian Rupees (INR) 66 and INR 85 more per member on healthcare over a 15-day reference period, than controls and additional expenditures (per member) incurred on inpatient care by cancer-affected households annually is equivalent to 36% to 44% of annual household expenditures of matched controls. Members without cancer in cancer-affected households used less health-care and spent less on healthcare. Overall, adult workforce participation rates were lower by between 2.4 and 3.2 percentage points compared to controls; whereas workforce participation rates among adult members without cancer were higher than in control households. Cancer-affected households also had significantly higher rates of borrowing and asset sales for financing outpatient care that were 3.3% to 4.0% higher compared to control households; and even higher for inpatient care.

## Introduction

The growth story of India has attracted much attention from economists, particularly over the last decade where annual average growth rates of income per capita have averaged 5.7%. On average, an Indian enjoyed an income of US$ 840 in 2011, nearly 5 times as high as his or her counterpart in 1960. Health gains have accompanied improved economic outcomes. In India, life expectancy at birth increased from 39 years in 1950 to 65 years in 2010, an increase of almost 67%. Even as these health and economic gains are being experienced, however, Indian health policymakers are faced with the challenge of non-communicable chronic diseases (NCD). According to the Global Burden of Disease (GBD) Study, ischemic heart disease, diabetes, stroke and asthma collectively accounted for almost 18% of years of life lost due to premature death in India in 2010, almost double their share in 1990 [1].

Although not immediately apparent from the GBD study, India also faces a major non-communicable disease burden from cancer. In India, each year nearly 600,000 cancer deaths occur [2], [3]; and 5-year cancer prevalence among individuals aged 15 years and over was estimated to be 1.7 million in 2008 [4]. Cancer incidence is increasing, owing to a mix of risk factors such as changes in diet and lifestyle, the legacy of high tobacco consumption along with population aging with cancer being more common in older populations [5], [6]. Cancers carry high levels of mortality and disability and require expensive treatments. Recent studies conclude that the aggregate economic impacts of cancer range from US$ 290 billion to US$ 900 billion worldwide [7]–[9].

This study assesses the economic burden of cancer on households in India using a large cross-sectional household dataset from India. This burden can be potentially significant in India, given that low public sector allocations to health (ranging from between 0.9% to 1.2% of GDP over the last few decades) and limited insurance options have forced households to rely on out of pocket spending to finance their healthcare [10]. We assess several dimensions of this burden, including health-care utilization, out-of-pocket expenditure on inpatient and outpatient care, aggregate household expenditures on items other than healthcare, reliance on borrowing and asset sales, and labor force participation rates of household members. We also examine the implications for other household members when an individual with cancer lives in a household, including their labor force participation, healthcare use and health spending. To our knowledge, this is the first systematic study of the economic burden of cancers on households in a developing country.

## Methodology

### 1. Statistical Methods

Previous calculations of the economic impacts of cancer cannot adequately describe household burden of cancer, for three reasons. First, their assessments of the economic burden of cancer are confounded by competing risks and co-morbidities. Because there are common risk factors that increase one’s risk of multiple diseases (e.g. tobacco use or obesity that could be linked to cancer, heart disease or diabetes), unadjusted estimates of the economic burden attributed to any one condition are likely to be upwardly biased. Second, socioeconomic and demographic differences across households can influence healthcare expenditures and other coping strategies in response to illness. Finally, aggregate calculations also include impacts of cancer on households unaffected by cancer, such as via an overall slowing of economic growth that lowers employment rates and incomes [7].

For these reasons, we focus specifically on households containing a member reporting as being diagnosed with cancer, i.e., ‘cancer-affected’ households, and compare their economic outcomes with households with similar socioeconomic and demographic characteristics that do not include a member with cancer. Matching on observable household characteristics allows selection of control households that are similar in socioeconomic characteristics and demographic composition to the sample of households affected with cancer.

We used propensity score matching (PSM) to compare the outcomes for a household containing a person with cancer to a matched household with no cancer case [11]. This generally consists of two stages. In the first stage, the probability that a household contains a member with cancer (the ‘propensity score’) is predicted based on household socioeconomic and demographic characteristics. This (pre-processing) stage involves the estimation of a logit or probit model. In this paper the first stage consisted of estimating the following logit model:
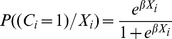
Here *C_i_* indicates whether household *i* contains a member with cancer. The vector *X_i_* indicates household demographic and socioeconomic characteristics, and *β* is a vector of the parameters to be estimated. In the second stage, cancer-affected households were matched to control households with similar propensity scores using STATA, version 12.0. For balance checking, for each covariate used in the regression model that generated the propensity scores, we compared the means between the cancer-affected households and matched control households using a *t*-test.

### 2. Main Source of Data

This paper is based on anonymized survey data collected by the National Sample Survey organization (NSSO), a department of the Indian Ministry of Statistics and Programme Implementation. We have sought and obtained permission from the NSSO to use this data in our research. This cross-sectional survey collected nationally representative data on morbidity and health care utilization in 2004 and covered nearly 74,000 households (383,000 individuals of all ages). The sampling was based on a multi-stage stratified design [12]. In India, NSSO health surveys are conducted roughly once a decade, with the last being in the year 2004. These surveys are developed with extensive pilot testing and constitute the most reliable source of national-level information on household healthcare utilization and expenditures. Information on socioeconomic and demographic characteristics, insurance status, healthcare use, medical and non-medical spending, sources of healthcare financing and work status for all household members was collected in person from a single key adult respondent.

### 3. Definition of Cancer-Affected Household

A household was defined as ‘cancer-affected’ if at least one of its members was: (1) currently living with cancer; or (b) hospitalized due to cancer in the year preceding the survey, whether or not currently alive. This definition included most diagnosed cancers, with the exception of those who died from cancer in the year preceding the survey without being hospitalized. In our survey data, about 54% of the cancer cases were reported as hospitalized in the year preceding the survey, which is less than the annual hospitalization rates (of around 70%) reported for cancer patients in developed countries such as Australia for which similar data are available [13]. Control households did not meet this definition.

### 4. Matching Variables

Cancer-affected households and control households were matched on several indicators, including educational status of the head of household, house-type, land ownership, water source, sanitation type, major source of livelihood, demographic structure (number of children, young adults and the elderly, proportion of household members that is female), caste (whether ‘scheduled caste or tribe’ or not, or ‘other backward caste’ or not), religion, rural/urban status and whether a member of the household was covered by social or private insurance. Dummies for 71 NSS sub-areas of residence were also included.

### 5. Outcome Variables

#### Healthcare utilization and expenditure of households

We used several outcome variables for healthcare utilization and spending. Healthcare utilization variables included hospital admissions per household member in the one year preceding the survey, length of hospital stay (in days) per household member, and outpatient visits per household member in the 15 days preceding the survey. Health expenditure variables included household out-of-pocket (OOP) expenditure per member for outpatient care in the 15 days preceding the survey, and household spending per member for hospital (inpatient) care in the one year preceding the survey.

#### Household non-Medical consumption spending

Treatment expenses and loss of income associated with cancer are likely to influence spending on items other than healthcare in the cancer-affected household [14]. Household consumption spending, *net* of healthcare expenditures, per member in the 15-day preceding the survey was used to capture this effect.

#### Financing of out-of-Pocket healthcare spending

To assess the degree of illness-related financial stress faced by households, we inquired whether the household borrowed funds or sold assets to support out of pocket spending on health care. Two indicator variables were used to capture this: whether a household borrowed funds or sold assets to finance inpatient (in the one year preceding the survey) and whether a household borrowed or sold assets to finance outpatient care in the 15 days preceding the survey [15]–[16].

#### Work force participation among adults and children

We compared adult workforce participation rates (the number of members aged 15 years and over who are currently working, divided by all members in the household aged 15 years and over) among cancer-affected household members and members of matched control households. A similar measure was constructed for comparing workforce participation rates among children aged 5–14 years of cancer-affected and control households.

#### Implications for members without cancer in cancer-Affected households

In a cancer-affected household, the burden of cancer might be felt in the form of changed workforce participation, healthcare spending and non-medical consumption of members without cancer, or in the ways healthcare is financed [17]–[20]. Therefore, we compared household-level outcomes (per person outpatient care visits, hospital admissions and health spending) of members without cancer in cancer-affected households and control households. We also compared healthcare utilization for ‘non-major conditions’ (specifically excluding stroke, diabetes, cancer, CVD and injuries) for the two sets of matched households.

### 6. Subgroup Analysis by Socioeconomic Status

Low levels of insurance coverage might lead to the economic burden of cancer being felt differentially across households of different economic status. The survey did not collect information on indicators of wealth or income. Instead, we used the education status of the head of household measured by years of schooling completed, as a proxy for household socioeconomic status (SES) [21]. To assess whether the economic burden varied with educational attainment of head of household, we conducted additional matching analyses comparing cancer-affected and control households separately for two subsets of population: one consisting of households where the educational status of the head of household was above the median (high SES households), and one of households where the educational status of the head of household was below or equal to the median (low SES) for the full set of households.

### 7. Robustness Checks

Several robustness checks were carried out by assessing results from alternative matching methods such as nearest neighbor and stratification; by estimating results after excluding from the data the 1% of households with the most out-of-pocket expenditures on cancer inpatient treatment to lower the risk of a few outliers influencing the results; and by excluding any household with at least one death because differential underreporting of deaths in household surveys. Further, to address possible risks of upward bias in estimates from using hospitalized cancer cases in our definition of cancer-affected households, we estimated a separate set of results when generating propensity scores by including an indicator of hospitalization in the propensity score equation.

## Results

Tables 1 and 2 present summary statistics for households with cancer, unmatched control households and matched control households (using nearest neighbor matching). Table 1 reveals that many of the means of the healthcare use and out-of-pocket spending indicators for matched cancer-affected and control households were closer to each other than to unmatched controls. Thus, a simple comparison of cancer households to a random selection of households not reporting cancer may yield upwardly biased estimates of the economic burden of cancer. Across a wide range of household socioeconomic and demographic characteristics used for matching, the sample means of matched (control) households were considerably closer to those of cancer-affected households than their unmatched counterparts (Table 2): *t*-tests for differences in sample means of cancer-affected and control households in the matched dataset (after nearest neighbor matching) showed no statistically significant differences at the 5% level. Column 5 of Table 2 shows the relevant t-statistic and the associated *p*-value (in parentheses) for the tests.

**Table 1:** Summary of Outcome Variables by Cancer-Affected or Control Households.

Outcome Variable (reference period)	Cancer-Affected Households	Control Households – Matched	Control Households – Unmatched
*Healthcare utilization*			
Hospital admissions, per household member (1 year)	0.275 (0.26, 0.29)	0.110* (0.10, 0.12)	0.098 (0.097, 0.099)
Public hospital admissions, per household member (1 year)	0.130 (0.12, 0.14)	0.048* (0.04, 0.06)	0.046 (0.0447, 0.0465)
Length of hospital stay (in days), per household member (1 year)	4.349 (3.84, 4.86)	1.134* (0.91, 1.36)	0.940 (0.916, 0.965)
Outpatient visits, per household member (15 days)	0.201 (0.18, 0.22)	0.145* (0.13, 0.16)	0.121 (0.120, 0.123)
Outpatient visits of non-cancer patients, per household member (15 days)	0.112 (0.10, 0.13)	0.145* (0.13, 0.16)	0.120 (0.119, 0.122)
Outpatient visits for non-major conditions, per household member (15 days)	0.073 (0.06, 0.08)	0.120* (0.10, 0.14)	0.098 (0.096, 0.099)
*Consumption*			
Inpatient OOP expenditure (INR), per household member (1 year)	5311 (4514, 6108)	1,079* (702, 1457)	764 (733, 794)
Outpatient OOP expenditure (INR), per household member (15 days)	118.33 (92.10, 144.56)	45.16* (33.94, 56.38)	34.16 (33.01, 35.30)
Outpatient OOP expenditure (INR) on non-major health conditions,per household member (15 days)	15.54 (11.16, 19.92)	33.49* (24.18, 42.80)	24.34 (23.46, 25.22)
Outpatient OOP expenditure (INR) for members without cancer,per household member (15 days)	28.03 (26.20, 35.87)	45.16* (33.94, 56.38)	33.14 (32.03, 34.24)
Non-medical consumption expenditure (INR), per household member (15 days)	294 (252, 337)	321 (301, 342)	322 (319, 324)
Percentage of households reporting borrowing or selling assetsto finance inpatient care (1 year)	51.40 (47.98, 54.82)	15.77* (13.28, 18.26)	15.72 (15.46, 15.98)
Percentage of households reporting borrowing/selling assets tofinance outpatient care (15 days)	6.46 (4.78, 8.14)	3.11* (1.92, 4.30)	2.45 (2.34, 2.56)
*Workforce Participation*			
Percentage of household members aged 15+ who are working	48.50 (46.69, 50.31)	50.90 (49.11, 52.69)	53.79 (53.59, 53.99)
Percentage of household members without cancer aged 15+ who are working	52.58 (50.30, 54.86)	50.90 (49.11, 52.69)	53.84 (53.64, 54.04)
Number of observations	821	824	72,582

*Note*: Estimates are based on calculations by authors using raw data from National Sample Survey data for 2004. Reference period refer to a household report in the period (15 days or 1 year) immediately preceding the survey. The data presented refer to all households, regardless of whether there was a death in the household. 95% confidence intervals are reported in parentheses underneath the means for each statistic.

* indicates that the treatment and matched controls are significantly different at the 5% level.

**Table 2:** Summary of Matching Variables by Cancer-Affected or Control Households.

Matching Variable	Cancer-Affected Households	Control Households– Matched	Control Households – Unmatched	*t*-statistic
Percentage rural households (%)	63.22 [59.91, 66.52]	61.88 [58.56, 65.19]	63.91 [63.56, 65.26]	0.56 (0.58)
Age structure				
Percentage of members aged 0–14 years (%)	25.49 [24.02, 26.95]	25.29 [23.77, 26.81]	28.42 [28.25, 28.59]	0.18 (0.86)
Percentage of members aged 15–29 years (%)	27.25 [25.79, 28.72]	27.92 [26.31, 29.52]	26.50 [26.33, 26.68]	−0.55 (0.59)
Percentage of members aged 60+ (%)	11.72 [10.36, 13.08]	11.56 [10.20, 12.93]	12.16 [12.00, 12.32]	0.16 (0.87)
Level of schooling of household head				
Percentage Illiterate (%)	31.32 [29.41, 33.23]	32.58 [30.53, 34.63]	38.67 [38.44, 38.91]	−0.87 (0.38)
Percentage with primary schooling (%)	29.60 [27.83, 31.37]	29.20 [27.44, 30.96]	29.25 [29.06, 29.44]	0.32 (0.75)
Percentage with secondary schooling (%)	9.87 [8.79, 10.94]	10.02 [8.80, 11.23]	7.65 [7.54, 7.76]	−0.18 (0.86)
Percentage with a graduate degree (%)	6.89 [5.78, 8.00]	5.90 [4.80, 7.00]	5.13 [5.02, 5.23]	1.24 (0.21)
Percentage female (%)	50.25 [49.16, 51.34]	50.42 [49.12, 51.72]	49.07 [48.93, 49.20]	−0.20 (0.85)
Energy source, drinking water and sanitation				
Percentage of households using cooking gas (%)	34.35 [31.10, 37.60]	35.69 [32.42, 38.96]	28.58 [28.25, 28.90]	−0.57 (0.57)
Percentage of households with piped water access (%)	41.66 [38.28, 45.03]	41.66 [38.29, 45.02]	41.71 [41.35, 42.07]	0.00 (0.99)
Percentage of households with latrine with septic tank (%)	38.98 [35.64, 42.31]	39.71 [36.36, 43.05]	33.32 [32.98, 33.67]	−0.30 (0.76)
Percentage of households with covered drainage (%)	20.10 [17.35, 22.84]	18.88 [16.21, 21.55]	17.44 [17.16, 17.72]	0.62 (0.53)
Caste				
Percentage scheduled caste/tribe households (%)	22.53 [19.67, 25.39]	22.66 [19.80, 25.52]	28.48 [28.15, 28.81]	−0.06 (0.95)
Percentage ‘other’ backward caste households (%)	39.22 [35.88, 42.56]	39.22 [35.88, 42.56]	37.55 [37.19, 37.90]	0.00 (0.99)
Religion				
Percentage Hindu (%)	81.49 [78.83, 84.14]	83.19 [80.54, 85.75]	79.44 [79.15, 79.74]	−0.81 (0.37)
Percentage Muslim (%)	12.06 [9.83, 14.29]	10.35 [8.27, 12.43]	11.81 [11.58, 12.04]	1.09 (0.27)
Percentage self-employed (%)	24.48 [21.54, 27.43]	24.48 [21.54, 27.42]	24.16 [23.85, 24.48]	0.00 (0.99)
Percentage insured (%)	3.41 [2.17, 4.65]	3.41 [2.17, 4.65]	2.20 [2.10, 2.31]	0.00 (0.99)
Geographic region				
Percentage in northern region (%)	12.42 [10.17, 14.68]	13.64 [11.30, 15.99]	10.35 [10.13, 10.57]	−0.74 (0.46)
Percentage in western region (%)	15.10 [12.65, 17.55]	14.13 [11.75, 16.51]	16.23 [15.96, 16.50]	0.56 (0.58)
Percentage in southern region (%)	26.67 [23.65, 29.70]	27.41 [24.36, 30.45]	23.74 [23.43, 24.05]	−0.33 (0.74)
Percentage in eastern region (%)	19.61 [16.89, 22.33]	18.88 [16.21, 21.55]	19.08 [18.79, 19.36]	0.37 (0.71)
Percentage in central region (%)	19.49 [16.78, 22.20]	19.49 [16.78, 22.19]	19.11 [18.82, 19.39]	0.00 (0.99)
Number of observations	821	824	72,582	1,645

*Note:* Estimates are based on calculations by authors using raw data from National Sample Survey data for 2004. The data presented refer to all households, regardless of whether there was a death in the household. Propensity score calculations used 71 NSS sub-region dummies rather than geographic region. The *t*-test reported in column 5 refers to a comparison of means between matched cancer-affected and control households; *p*-values are reported in parentheses below the *t*-statistic. *indicates that the treatment and matched controls are significantly different at the 5% level.

Columns 2 through 5 of Table 3 present results for healthcare utilization under multiple specifications: a direct comparison of cancer-affected and control households using the nearest neighbor and stratification approaches (columns 2 and 3), a comparison that excluded all households experiencing at least one death in the preceding year, and a third that excluded 1% of the households with the highest levels of cancer-related inpatient spending. Cancer-affected households experience an extra 15.8 to 17.3 hospital admissions per 100 members annually, and an extra 5.6 to 7.6 outpatient visits per 100 members in the 15 days preceding the survey, compared to matched control households. Cancer-affected households also reported more days spent in hospitals per member. Per person outpatient visits of members without cancer (in cancer-affected households) were lower – by between 2 and 3 visits for every 100 members – than for household members in matched controls in the 15 days preceding the survey. Per person outpatient visits for non-major health conditions in the 15-days preceding the survey were also lower in cancer-affected households, by between 2.8 and 4.7 visits per 100 members, compared to matched controls.

**Table 3:** Effects of Cancers on Household Healthcare Utilization in India.

Outcome Indicator (reference period)	All Matched Households (by nearest neighbor)	All Matched Households (by stratification)	Excluding Households with Death (by nearest neighbor)	Excluding Households with 1% Most Expensive Cancer Cases (by nearest neighbor)
Hospital admissions, per household member (1 year)	0.165 (<0.01)	0.173 (<0.01)	0.165 (<0.01)	0.158 (<0.01)
Public hospital admissions, per household member (1 year)	0.082 (<0.01)	0.085 (<0.01)	0.074 (<0.01)	0.081 (<0.01)
Length of hospital stay, per household member (1 year)	3.215 (<0.01)	3.378 (<0.01)	3.323 (<0.01)	3.303 (<0.01)
Outpatient visits, per household member (15 days)	0.056 (<0.01)	0.069 (<0.01)	0.076 (<0.01)	0.057 (<0.01)
Public outpatient visits, per household member (15 days)	0.029 (<0.01)	0.035 (<0.01)	0.031 (<0.01)	0.025 (<0.01)
Outpatient visits of non-cancer patients per householdmember (15 days)	−0.033 (<0.01)	−0.021 (0.013)	−0.018 (0.146)	−0.031 (0.01)
Outpatient visits for non-major health conditions,per household member (15 days)	−0.047 (<0.01)	−0.032 (<0.01)	−0.028 (<0.01)	−0.039 (<0.01)
Number of treated (and matched control) observations	821 (824)	821 (71,761)	735 (743)	813 (817)

*Notes*: INR = Indian Rupees. Values in parentheses refer to *p*-values that the matched cancer-affected and control outcomes differ in a two-tailed test. ‘Non-major’ health conditions refer to all health conditions except cancer, heart disease, stroke, injuries & diabetes.

Columns 2 through 5 of Table 4 present results on household consumption and labor force participation. Out-of-pocket health expenditures are significantly higher in households with cancer compared to controls, by between INR 3,576 and INR 4,438 per member for inpatient expenditures in the year preceding the survey and by between INR 66 and INR 85 per member in the 15 days preceding the survey per person for outpatient visits. Expenditures on non-major health conditions and on healthcare for individuals other than the person with cancer in cancer-affected households were lower, relative to controls. Spending per household member on non-medical consumption was lower in cancer-affected households than matched controls, by amounts between INR 27 and INR 85 per household member in the 15 days preceding the survey, although the differences were not always statistically indistinguishable from zero.

**Table 4:** Effects of Cancers on Household Consumption and Workforce Participation in India.

Outcome Indicator (reference period)	All Matched Households (by nearest neighbor)	All Matched Households (by stratification)	Excluding Households with Death (by nearest neighbor)	Excluding Households with 1% most expensive cancer cases (by nearest neighbor)
*Household Consumption*				
Inpatient OOP expenditure (INR), per household member (1 year)	4,232.08 (<0.01)	4,437.66 (<0.01)	4,044.30 (<0.01)	3,575.74 (<0.01)
Outpatient OOP expenditure (INR), per household member (15 days)	73.18 (<0.01)	78.91 (<0.01)	85.31 (<0.01)	65.68 (<0.01)
Outpatient OOP expenditure (INR) on non-major health conditions,per household member (15 days)	−17.95 (<0.01)	−12.80 (<0.01)	−10.99 (<0.01)	−11.99 (<0.01)
Outpatient OOP expenditure (INR) for members without cancer,per household member (15 days)	−17.13 (0.01)	−11.40 (<0.01)	−10.55 (0.05)	−13.78 (0.03)
Non-medical consumption expenditure (INR), per householdmember (15 days)	−26.69 (0.265)	−49.75 (0.020)	−84.60 (0.033)	−32.15 (0.174)
Percentage of households reporting borrowing or selling assetsto finance inpatient care (1 year)	0.356 (<0.01)	0.361 (<0.01)	0.392 (<0.01)	0.335 (<0.01)
Percentage of households reporting borrowing/selling assetsto finance outpatient care (15 days)	0.033 (<0.01)	0.040 (<0.01)	0.039 (<0.01)	0.033 (<0.01)
*Workforce Participation*				
Percentage of household members aged 15+ who are working	−2.40 (0.07)	−3.00 (<0.01)	−2.80 (<0.06)	−3.20 (0.02)
Percentage of household members without cancer aged 15+ whoare working	1.70 (0.26)	1.10 (<0.36)	1.90 (0.26)	0.80 (0.59)
Percentage of household members aged 5–14 years who are working	−0.30 (0.50)	−0.30 (0.32)	0.01 (0.91)	−0.10 (0.88)
Number of treated (and matched control) observations	821 (824)	821 (71,761)	735 (743)	817(813)

*Notes*: INR = Indian Rupees. Values in parentheses refer to *p*-values that the matched cancer-affected and control outcomes differ in a two-tailed test. ‘Non-major’ health conditions refer to conditions other than cancer, heart disease, stroke, injuries and diabetes.

Cancer-affected households rely to a significantly greater extent on borrowing or asset sales for financing out-of-pocket spending on treatment than matched controls: between 33.5% and 39.2% for inpatient spending in the year preceding the survey; and between 3.3% to 4.0% for outpatient spending in the 15-days preceding the survey. Current workforce participation rates among household members aged 15 years and above are lower by between 2.4% and 3.2% for households with cancer relative to matched controls. Workforce participation rates were higher among adult members in cancer-affected households when the individual with cancer was *excluded* from consideration – by between 0.80% and 1.90% – but the results are not statistically significant. Differences in work-force participation rates among children (aged 5–14 years) between cancer-affected and control households were also statistically indistinguishable from zero and varied from 0.01% to −0.30% in absolute magnitude.

We also conducted a separate set of analyses, in which an indicator of hospitalization was included as a matching variable to generate propensity scores (in addition to the core socioeconomic and demographic characteristics). Consequently, a large number of households whose members were hospitalized for reasons other than cancer were included as controls and lowered estimates of the economic impacts due to cancer. However, as indicated in Tables 5 and 6, although our estimates of the economic burden on households are lower, they do not affect the basic direction of our conclusions.

**Table 5:** Robustness Check – Effects of Cancers on Healthcare Utilization in India.

Outcome Indicator (reference period)	All Matched Households (by nearest neighbor)	All Matched Households (by stratification)	Excluding Households with Death (bynearest neighbor)	Excluding Households with 1% most expensive cancer cases (by nearest neighbor)
Hospital admissions per household member (1 year)	0.045 (<0.01)	0.044 (<0.01)	0.030 (0.013)	0.041 (<0.01)
Public hospital admissions per household member (1 year)	0.021 (0.03)	0.027 (<0.01)	0.018 (0.058)	0.023 (0.017)
Length of hospital stay per household member (1 year)	2.059 (<0.01)	2.142 (<0.01)	2.028 (<0.01)	2.013 (<0.01)
Outpatient visits per household member (15 days)	0.057 (<0.01)	0.052 (<0.01)	0.056 (<0.01)	0.036 (<0.01)
Public outpatient visits per household member (15 days)	0.027 (<0.01)	0.025 (<0.01)	0.032 (<0.01)	0.028 (<0.01)
Outpatient visits of non-cancer patients per householdmember (15 days)	−0.033 (<0.01)	−0.037 (<0.01)	−0.037 (<0.01)	−0.051 (<0.01)
Outpatient visits for non-major health conditions, per household member (15 days)	−0.040 (<0.01)	−0.040 (<0.01)	−0.046 (<0.01)	−0.052 (<0.01)
Number of Treated (and Matched Control) Observations	821 (801)	821 (71,555)	735 (725)	813 (800)

*Notes*: Robustness check refers to propensity scores generated with a hospitalization. INR = Indian Rupees. Values in parentheses refer to *p*-values that the matched cancer-affected and control outcomes differ in a two-tailed test. ‘Non-major’ health conditions refer to conditions excluding cancer, heart disease, stroke, injuries & diabetes.

**Table 6:** Robustness Check – Effect of Cancers on Household Consumption and Workforce Participation in India.

Outcome Indicator (reference period)	All Matched Households (by nearest neighbor)	All Matched Households (by stratification)	Excluding Households with Death (by nearest neighbor)	Excluding Households with 1% most expensive cancer cases (by nearest neighbor)
*Household Consumption*				
Inpatient OOP expenditure (INR), per household member (1 year)	3,403.94 (<0.01)	3,401.52 (<0.01)	2,942.23 (<0.01)	2,534.72 (<0.01)
Outpatient OOP expenditure (INR), per household member (15 days)	72.14 (<0.01)	66.31 (<0.01)	75.42 (<0.01)	49.50 (<0.01)
Outpatient OOP expenditure (INR) on non-major health conditions, per household member (15 days)	−16.58 (<0.04)	−20.82 (<0.01)	−18.02 (<0.01)	−29.67(<0.01)
Outpatient OOP expenditure (INR) for members without cancer, per household member (15 days)	−18.16 (<0.06)	−23.95 (<0.01)	−29.96 (<0.01)	−19.95 (<0.01)
Non-medical consumption expenditure (INR), per household member (15 days)	−16.91 (0.509)	−36.27 (0.094)	−44.23 (0.089)	−37.16 (0.164)
Percentage of households reporting borrowing or selling assets to finance inpatient care (1 year)	0.171 (<0.01)	0.169 (<0.01)	0.154 (<0.01)	0.137 (<0.01)
Percentage of households reporting borrowing/selling assets to finance outpatient care (15 days)	0.028 (0.01)	0.032 (<0.01)	0.035 (<0.01)	0.020 (<0.072)
*Workforce Participation*				
Percentage of household members aged 15+ who are working	−0.50 (0.683)	−1.80 (0.051)	−2.40 (0.088)	−2.00 (0.129)
Percentage of household members without cancer aged 15+ whoare working	3.60 (0.018)	2.30 (0.052)	2.20 (0.174)	2.00 (0.168)
Percentage of household members aged 5–14 years who are working	−0.60 (0.18)	−0.30 (0.27)	−0.00 (0.96)	−0.30 (0.46)
Number of treated (and matched control) observations	821 (801)	821 (71,555)	735 (725)	813 (800)

*Notes*: Robustness check refers to propensity-score matching that included hospitalization. INR = Indian Rupees. Values in parentheses refer to *p*-values that the matched cancer-affected and control outcomes differ in a two-tailed test. ‘Non-major’ health conditions refer to conditions other than cancer, heart disease, stroke, injuries and diabetes.

As part of a subgroup analysis, Table 7 presents results for analyses in which the population is divided into two sub-groups by education of household head: education greater the median (high SES) and below or equal to the median (low SES). Comparisons between the two groups are thus based on outcomes relative to their (respective) matched controls. In general, both high and low SES cancer-affected households experienced more hospital admissions, more days in hospitals and increased outpatient visits. However, high SES households experienced a greater increase in private hospital admissions, and a smaller increase in outpatient visits (per member) relative to matched controls. Indeed, high SES cancer-affected households experienced a decline in outpatient visits among members without cancer. This was not observed among low-SES households: and almost the entire difference in the change in overall number of outpatient visits among the two groups can be explained by the declining outpatient use by members without cancer in high SES households.

**Table 7:** Burden of Cancer by Educational Status of Head of Household, 2004.

Outcome Indicator (reference period)	By Education of Household Head	
	Above Median(low SES)	Below or Equal to Median (high SES)
*Healthcare Utilization*		
Hospital admissions, per household member (1 year)	0.160 (<0.01)	0.168 (<0.01)
Public hospital admissions, per household member (1 year)	0.067 (<0.01)	0.090 (<0.01)
Length of hospital stay, per household member (1 year)	3.412 (<0.01)	3.355 (<0.01)
Outpatient visits, per household member (15 days)	0.047 (0.02)	0.089 (<0.01)
Public outpatient visits, per household member (15 days)	0.026 (<0.01)	0.047 (<0.01)
Outpatient visits of non-cancer patients per household member (15 days)	−0.034 (0.09)	−0.007 (0.61)
Outpatient visits for non-major health conditions, per household member (15 days)	−0.027 (0.06)	−0.026 (0.01)
*Household Consumption*		
Inpatient OOP expenditure (INR), per household member (1 year)	6,127.36 (<0.01)	3,223.36 (<0.01)
Outpatient OOP expenditure (INR), per household member (15 days)	79.32 (0.02)	69.90 (<0.01)
Outpatient OOP expenditure (INR) on non-major health conditions, per household member (15 days)	−36.04 (<0.01)	−1.15 (0.75)
Outpatient OOP expenditure (INR) for members without cancer, per household member (15 days)	−40.52 (0.04)	1.59 (0.76)
Non-medical consumption expenditure (INR), per household member (15 days)	−63.50 (0.22)	−40.80 (0.02)
Percentage of households reporting borrowing or selling assets to finance inpatient care (1 year)	0.311 (<0.01)	0.426 (<0.01)
Percentage of households reporting borrowing/selling assets to finance outpatient care (15 days)	0.003 (0.83)	0.071 (<0.01)
*Workforce Participation*		
Percentage of household members aged 15+ who are working	−1.60 (0.40)	−3.80 (0.04)
Percentage of household members without cancer aged 15+ who are working	1.50 (0.50)	1.10 (0.59)
Percentage of household members aged 5–14 years who are working	0.20 (0.59)	−0.40 (0.65)
Number of treated (and matched control) observations	467 (470)	353 (354)

*Note*: Authors’ estimates using a dataset consisting of only matched households using the nearest neighbor method. Values in parentheses refer to *p*-values.

After accounting for controls, high SES households also spend more per member on inpatient and outpatient care compared to low SES cancer-affected households. The former also spend less out of pocket on the healthcare of members without cancer, and on non-major health conditions; and they borrowed less compared to low SES cancer-affected households. Among high SES cancer-affected households, adult labor force participation is lower than matched controls but statistically indistinguishable from zero. Low SES cancer-affected households experienced lower adult labor force participation relative to controls, with the difference also being larger in absolute magnitude when compared to adult workforce participation among high SES households. Finally, high SES households also see a statistically insignificant difference in non-medical consumption relative to controls (the decline is 14.5% of non-medical expenditures of matched controls). Low SES households experienced lower non-medical consumption that is both statistically significant and slightly larger as a share of non-medical expenditures of matched controls (16.4%).

## Discussion

As expected, cancer-affected households experience a greater number of hospital admissions (inpatient stays) and outpatient visits, compared to matched controls. Given the low population coverage of health insurance in India and a poorly run public sector, it is not surprising to find a large burden of out-of-pocket spending on households affected by cancer [22]. The additional expenditures (per member) incurred on inpatient care by cancer-affected households annually is equivalent to 36% to 44% of annual household expenditures of matched controls (of INR 9,988). Roughly 34% to 42% percent of all spending (INR 15,343 annually) by an average cancer-affected household is for out-of-pocket treatment inpatient and outpatient expenses. Households with higher SES spend more on healthcare out of pocket as a percentage of total spending (60%) relative to their low SES counterparts (53%).

Out-of-pocket expenses associated with treatment for cancer and any loss of income affect expenditures of a cancer-affected household on non-medical consumption. We would expect household non-medical consumption be lower when some members have cancer, unless the household was able to effectively insure against associated financial risks [14]–[15]. We find that cancer-affected households have lower expenditures per person. The estimated adverse impacts on consumption spending per person net of health spending ranged between INR 649 to INR 2,058 annually, indicating that Indian households are unable to fully protect themselves against financial risks from cancer, relative to controls. However, this decline is considerably less than the additional estimated inpatient out-of-pocket expenditures (INR 3,576 to INR 4,438). This conclusion also holds for households with different SES. Higher SES cancer-affected households experience lower (by INR 1,545) non-medical expenses and higher inpatient care out of pocket expenses of INR 6,127, relative to matched controls. Similarly low SES households had non-medical expenses that were INR 992 less than matched controls, but higher annual inpatient expenses of INR 3,223. Thus households are relying on other ways to protect their non-health spending against cancer-related expenditures.

Households also cope with the costs of cancer by increasing the burden on other members on unaffected members [14], [17]–[20], [23]. Outpatient visits and out-of-pocket healthcare spending per member were both significantly lower in cancer-affected households compared to matched controls, once the individual with cancer was excluded from the analysis. However, when data are broken down by SES, this conclusion holds only for higher SES households. We also compared healthcare utilization and expenditure for non-major conditions (i.e. other than cancer, stroke, heart disease, injuries and diabetes) on the assumption that the occurrence of a serious condition would lower healthcare use for the former. We found that per person outpatient care use for non-major conditions was lower by between 3 and 5 visits per 100 persons among cancer-affected households relative to matched controls, and this conclusion holds even if data are broken down into two SES groups. Expenditures on non-major health conditions were also lower, although in this case, the results are being driven primary by lower expenses among high SES households (Table 7). The lack of change in outpatient care use among members without cancer in low SES households likely reflects the already low outpatient care use among them, so further declines associated with cancer are unlikely to be significant.

Income losses from days missed at work by the sick person and their caregivers could arise because more than 90% of India’s workers are mostly employed in the informal sector with limited social security benefits. Overall, cancer-affected households have a lower labor force participation rate than matched controls of between 2.4 and 3.2 percentage points. However, workforce participation of members without cancer in cancer-affected households was 0.8 to 1.9 percentage points higher than control households, although the results are statistically indistinguishable from zero (see studies that analyzed workforce participation in other disease contexts [24], [25]). These estimates do not indicate impacts on hours of work or earnings, but suggest that the adverse impact of cancer on household earnings may be partly countered by rising workforce participation among healthier household members. Even here though, adult workforce participation declines much more in low SES households (relative to controls) than in high SES households. These ideas do not carry over, however, for child workers where the differences between cancer-affected households and matched controls are small and statistically insignificant, even when data are broken down by SES. One possible explanation is that most of the response in household economic activities associated with cancer occurs among adults. Alternatively, given that only 2.4% of the sample of children aged 5–14 years reported working in the survey, understanding the burden of cancer on children in the form of work participation and/or caregiving activities with any statistical precision may require collecting larger samples.

Households cope with increased requirements for health spending associated with cancer by borrowing or selling assets. However, the extent to which they rely on sales of assets and borrowing varies inversely with economic status: there is an almost 11 percentage point difference in household reliance on borrowing and sale of assets for financing inpatient care in high SES cancer-affected households, compared to low SES households. In sum, not only can cancer have long-term implications for household economic well-being if borrowing costs are high or if income earning assets are sold, but also it may exacerbate pre-existing economic inequalities. Our results also suggest a difference in the way households at different levels of SES respond to the economic challenges of cancer. Specifically, higher-SES households seem to adjust by lowering their spending on outpatient care for members without cancer and for non-major health conditions, along with some decline in non-medical expenses and reliance on borrowing/assets. Low SES households, however, have a narrower set of options: more borrowing and sale of assets and lower non-medical consumption.

Our study has multiple strengths. Matching cancer-affected households to ‘control’ households (not affected by cancer) on large set of observable socioeconomic and demographic characteristics reduces confounding that arises from non-random assignment of cancer. Our results suggest that the economic burden of cancer – be it in terms of public subsidies or out-of-pocket spending by households – may not be as large as one might conclude in the absence of matching. Moreover, we use a nationally representative household survey that contains detailed information on healthcare utilization and out-of-pocket expenditure on health services by individuals, the methods by which out-of-pocket spending was financed, along with information on individual-level workforce participation. Finally, our study findings rely on multiple checks for robustness. Our results hold up across different matching methods, as well as matching after excluding households with a death from any cause, and matching after excluding the top 1% households with the most out of pocket spending on cancer treatment.

There are limitations to our study. Our identification of cancer-affected households relies on self-reports, which may lead to inaccurate estimation of cancer cases. It is noteworthy though, that the prevalence of cancer cases in the survey data we used was 0.22%, which was very close to the prevalence estimate reported by GLOBOCAN (2008) of 0.23% among individuals aged 15-years or older.

From the health survey we used, there are an estimated 102 thousand deaths due to cancer in 2004, compared to 634 thousand deaths per GLOBOCAN (2008) estimates based on cancer registries in India, and about 560 thousand deaths in 2000 based on the Million Deaths Study [3], [4]. Underestimates of cancer deaths arise partly because 49% of deaths reported in the survey are not assigned a cause. However, another reason is that deaths (from any cause) are undercounted in NSS surveys. Cancer impact estimates could be biased downwards if healthcare utilization and expenditures are concentrated in the time immediately preceding death, and a disproportionate number of cancer deaths are excluded. On the other hand, a disproportionate share of cancer deaths included hospitalizations (95% of cancer cases resulting in deaths reported in the survey were hospitalized in the preceding 12 months) and this could bias estimates of the economic burden of cancer upwards given the definition of a cancer-affected household includes all households that had a member hospitalized due to cancer in the year preceding the survey. As noted earlier, estimated annual hospitalization rate for cancer in our survey population in cancer-affected households is not inordinately high at 54%, well below rates seen in a developed country with excellent prevalence and hospital separations data, e.g. 70% in Australia. However, we also addressed this issue directly by undertaking additional analyses limited to households that did not experience deaths (since the incidence of hospitalization was much higher in hospitalized cases), irrespective of cause. Although the absolute values of coefficient estimates are lower, the direction of the results is similar. It is noteworthy that if households with cancer deaths are excluded, we end up with an estimate of 0.93 million cancer cases from NSS data in 2004 (after using sampling weights) versus 1.07 million for GLOBOCAN (2008).

Matching methods cannot account for unobservable factors that drive household risks for cancer. For example, our 2004 data does not include information on tobacco and alcohol consumption, dietary history or obesity in the household. Nor do we have information on the occupational history of household members that could affect cancer risks. Unavailability of this information may lead to the exclusion of at-risk households from matched controls and can bias our estimated economic burden of cancer upwards if the excluded households are also at risk for acquiring other serious illnesses and increased health care use. Our matched control households reported higher levels of health care use and out of pocket spending compared to the average survey household, so this risk is lower. Separately, we tried to address this issue by including a hospitalization indicator in constructing propensity scores prior to matching. As a result, the estimated economic burden of cancer was lower, but the direction of the conclusions remained unchanged. To effectively address this subject would require longitudinal data, which the NSS surveys do not currently collect.

Biased estimates may also have resulted because we did not have information on differences in physical access (such as distance to health facilities) which can influence the diagnosis of cancer and any associated health spending. The absence of information on distance to health facilities from the propensity score equation could potentially cause an upward bias in our estimates of out of pocket spending and healthcare use if, as is likely, individuals with better access also happen to be wealthier on average. We sought to address this through the inclusion of three sets of variables in the propensity score equation, two of them being a range of controls of living conditions (access to drains, piped water, etc.) and rural residence that are likely to be positively correlated with physical access [26]. In addition, we used 71 indicators of location used by the NSSO to indicate sub-regions with varying economic and climatic characteristics and these may capture many region-specific differences related to physical access to health services.

The survey also did not collect information on the severity of cancer. If information on severity were available, we could have used a variation of the propensity score matching method for the multiple treatment case to assess the economic burden on households by severity of condition [27]. In the absence of this information, all we can estimate is the average burden for different levels of cancer severity. It is nevertheless possible that what we have in the data are relatively more severe cancer cases thereby what we are capturing is, in fact, the economic burden of cancer for the more severe cases.

Our estimates of the economic burden of cancer are for 2004, and can be expected to have risen considerably since then, both on account of general inflation as well on account of changes in the technology of cancer treatment. Information on the impact of the latter on treatment costs is unavailable for India. However, consumer price index data suggest that the treatment costs are likely to be at least 70% higher at present compared to 2004 [28].

### Conclusions

Our finding provides much better understanding of economic burden of cancer at the household level. To our knowledge, ours is the first paper to estimate this burden for households in a developing country. Our use of matching also helps to address potential confounding associated with socioeconomic and demographic differences in estimating the economic burden of cancer.

The major policy implication of our findings is the need for protection against the financial risks from cancer for Indian households. This is not surprising given the heavy reliance on out of pocket spending in financing healthcare in India: government financing accounts for only about one-fourth of India’s aggregate health spending of 4.5% of GDP, and most of the residual spending is in the form of out of pocket spending by households. Our results also show that households across the SES spectrum face a large economic risk from cancer, and the risk seems particularly serious for low SES households who rely on borrowing and asset sales to a much greater extent to finance their healthcare.

Unlike in 2004 the year our survey was conducted, over the last few years, a number of publicly funded health insurance schemes have emerged in India [29]. These schemes and existing subsidized public sector health facilities are likely to have provided improved protection against the economic costs of cancer. However, coverage is not comprehensive in many of these programs and non-poor households are often ineligible to benefit from such programs. For example, the Rashtriya Swasthya Bima Yojana (RSBY) is a fully government funded insurance scheme targeted only at the poor and provides coverage for hospital-based treatments including cancer, at both private and public healthcare facilities. Although the scheme now covers more than one hundred million people in India, the financial cover it provides is relatively small (INR 30,000) for a family of four and is inadequate given the financial costs of cancer treatment [30]. More generous publicly financed schemes are in place in a few Indian states such as Andhra Pradesh and Tamil Nadu, and they cover a broader category of households than just the poor but these comprise only a subset of India’s population and face issues of long-term financial sustainability.

Apart from the limited financial cover for the poor, a key conclusion of our paper is that higher SES households also face a serious financial risk from cancer. The problem of lack of coverage of the non-poor is exacerbated by various age-limits that exist for voluntary private insurance coverage in India, and a public sector that is underfunded and provides poor quality services for all population sub-groups.

These gaps in coverage necessitate thinking more broadly about pooling mechanisms than the strategy of just targeting the poor with publicly funded programs. However, expanded coverage for cancer (and other conditions requiring expensive treatment such as heart disease) is challenging in a resource constrained health sector such as in India as it competes with other pressing healthcare needs for the poor, including the needs for child survival, malaria and tuberculosis, as it charts out a path for achieving universal coverage over the next 10–15 years [31]. An interim mechanism may well be a regulatory environment that encourages private sector pooling to fund cancer care and expanded publicly funded cover for the poor for expensive to treat medical conditions. From a longer term perspective, mechanisms to lower the risk of acquiring cancer, including improved screening might also be prioritized.

## References

[1] Institute of Health Metrics and Evaluation (2013) GBD Profile: India. Available: http://www.healthmetricsandevaluation.org/sites/default/files/country-profiles. Accessed 2013 June 26.

[2] Ferlay J, Shin HR, Bray F, Forman D, Mathers C, et al (2010) Estimates of worldwide burden of cancer in 2008: GLOBOCAN 2008. International Journal of Cancer 127: 2893–2917. 21351269 10.1002/ijc.25516

[3] Dikshit R, Gupta PC, Ramasundarahettige C, Gajalakshmi V, Aleksandrowicz L, et al (2012) Cancer mortality in India: a nationally representative survey. Lancet 379: 1807–1816. 22460346 10.1016/S0140-6736(12)60358-4

[4] GLOBOCAN (2008) The GLOBOCAN project. Available: http://globocan.iarc.fr/. Accessed 2013 February 10.

[5] Popkin B, Horton S, Kim S, Mahal A, Shuigao J (2001) Trends in diet, nutritional status, and diet-related non-communicable diseases in China and India: the economic costs of the nutrition transition. Nutrition Reviews 59(12): 379–390. 11766908 10.1111/j.1753-4887.2001.tb06967.x

[6] Murthy N, Chaudhry K, Rath G (2008) Burden of cancer and projections for 2016, Indian scenario: gaps in the availability of radiotherapy treatment facilities. Asia Pacific Journal of Cancer 9: 671–677. 19256757

[7] Bloom D, Cafiero E, Jane-Llopis E, Abrahams-Gessel S, Bloom L, Fathima S, et al.. (2011) The Global Economic Burden of Non-Communicable Diseases. Boston, MA: Harvard School of Public Health and World Economic Forum.

[8] Abegunde DO, Mathers CD, Adam T, Ortegon M, Strong K (2007) The burden and costs of chronic diseases in low-income and middle-income countries. Lancet 370: 1929–1938. 18063029 10.1016/S0140-6736(07)61696-1

[9] John R, Ross H (2011) Global cancer facts & figures. 2nd edition. Atlanta: American Cancer Society.

[10] Reddy K, Patel V, Jha P, Paul V, Shiva Kumar AK, et al (2011) Towards achievement of universal health care in India by 2020: A call to action, The Lancet. 377: 760–8. 10.1016/S0140-6736(10)61960-5 PMC4991755 21227489

[11] Rosenbaum P, Rubin D (1983) The central role of the propensity score in observational studies for causal effects. Biometrika 70: 41–55.

[12] National Sample Survey Organization (NSSO) (2006) Morbidity, health care and the conditions of the aged, NSSO 60th Round (January-June 2004). New Delhi: National Sample Survey Organisation. Ministry of Statistics and Programme Implementation. Government of India.

[13] Australian Institute of Health and Welfare (2012) Australian Health Statistics 2010–11. Health Services Series no. 43, Category no. HSE 117. Canberra: Australia.

[14] Gertler P, Gruber J (2002) Insuring consumption against illness. American Economic Review 92(1): 51–70. 29058389 10.1257/000282802760015603

[15] Islam A, Maitra P (2012) Health shocks and consumption smoothing in rural households: Does microcredit have a role to play? Journal of Development Economics 97(2): 232–43.

[16] Wagstaff A (2008) Measuring financial protection in health. Policy Research Working Paper 4554. Washington, D.C.: The World Bank.

[17] Lilly M, Laporte A, Coyte P (2010) Do they care too much to work? The influence of caregiving intensity on the labour force participation of unpaid caregivers in Canada. Journal of Health Economics 29: 895–903. 20864197 10.1016/j.jhealeco.2010.08.007

[18] Ciani E (2011) Informal Adult Care and Caregivers’ Employment in Europe. Labour Economics 19: 155–164.

[19] Shaw W, Patterson T, Semple S, Ho S, Irwin M, et al (1997) Longitudinal analysis of multiple indicators of health decline among spousal caregivers. Annals of Behavioural Medicine 19: 101–109. 10.1007/BF02883326 9603684

[20] Roberts A (1992) The labor market consequences of family illness. Journal of Mental Health Economics and Policy 2: 183–195. 10.1002/(sici)1099-176x(199912)2:4<183::aid-mhp62>3.0.co;2-1 11967430

[21] Jolliffe D (1997) Whose education matters in the determination of household income? Evidence from a developing country. Discussion paper #39. Washington, D.C.: International Food Policy Research Institute, Food, Consumption and Nutrition Division.

[22] Mahal A (2010) Health financing in India. In: Mahal A, Debroy B and Bhandari L, editors, India Health Report. New Delhi: Business Standard Press. 109–126.

[23] Yamano T, Jayne T (2004) Measuring the impacts of working-age adult mortality on small-scale farm households in Kenya. World Development 32: 91–119.

[24] Harris A (2008) Chronic disease and labour force participation in Australia: an endogenous multivariate probit analysis of clinical prevalence data. Research paper no. 25/08. Clayton, Australia: Monash University, Centre of Health Economics.

[25] Passey ME, Shrestha RN, Bertram MY, Schofield DJ, Vos T, et al (2012) The impact of diabetes prevention on labour force participation and income of older Australians: an economic study. BMC Public Health 12: 16. 22225701 10.1186/1471-2458-12-16 PMC3295674

[26] Malhotra C, Kyung Do Y (2012) Socioeconomic disparities in health system responsiveness in India. Health Policy and Planning doi:10.1093/heapol/czs051. 10.1093/heapol/czs051 PMC3584994 22709921

[27] Morena-Serra R (2009) Health programme evaluation by propensity score matching: Accounting for treatment intensity and health externalities with an application to Brazil. Health Economics Group Working Paper 09/05, University of York, United Kingdom.

[28] Government of India (2012) Economic Survey 2011–12. New Delhi, India: Government of India, Ministry of Finance.

[29] Fan VY, Karan A, Mahal A (2012) State health insurance and out-of-pocket health expenditures in Andhra Pradesh, India. International Journal of Health Care Finance and Economics 12: 1–27. 22767078 10.1007/s10754-012-9110-5

[30] Ministry of Labour and Employment (2008) RSBY Guidelines: Guidelines13.3.08_rev.pdf (New), Annexure I. Available: http://www.rsby.gov.in/Documents.aspx?id=25/.Accessed 2013 June 30.

[31] John Jacob T, Dandona L, Sharma VP, Kakkar M (2011) Continuing challenge of infectious disease in India. The Lancet 377: 252–269. 10.1016/S0140-6736(10)61265-2 21227500

